# Family-based association tests for rare variants with censored traits

**DOI:** 10.1371/journal.pone.0210870

**Published:** 2019-01-25

**Authors:** Wenjing Qi, Andrew S. Allen, Yi-Ju Li

**Affiliations:** 1 Department of Biostatistics and Bioinformatics, Duke University, Durham, NC, United States of America; 2 Duke Molecular Physiology Institute, Duke University, Durham, NC, United States of America; 3 Center for Statistical Genetics and Genomics, Duke University, Durham, NC, United States of America; University of Texas Health Science Center at San Antonio, UNITED STATES

## Abstract

We propose a set of family-based burden and kernel tests for censored traits (FamBAC and FamKAC). Here, censored traits refer to time-to-event outcomes, for instance, age-at-onset of a disease. To model censored traits in family-based designs, we used the frailty model, which incorporated not only fixed genetic effects of rare variants in a region of interest but also random polygenic effects shared within families. We first partitioned genotype scores of rare variants into orthogonal between- and within-family components, and then derived their corresponding efficient score statistics from the frailty model. Finally, FamBAC and FamKAC were constructed by aggregating the weighted efficient scores of the within-family components across rare variants and subjects. FamBAC collapsed rare variants within subject first to form a burden test that followed a chi-squared distribution; whereas FamKAC was a variant component test following a mixture of chi-squared distributions. For FamKAC, *p*-values can be computed by permutation tests or for computational efficiency by approximation methods. Through simulation studies, we showed that type I error was correctly controlled by FamBAC for various variant weighting schemes (0.0371 to 0.0527). However, FamKAC type I error rates based on approximation methods were deflated (max 0.0376) but improved by permutation tests. Our simulations also demonstrated that burden test FamBAC had higher power than kernel test FamKAC when high proportion (*e*.g. ≥ 80%) of causal variants had effects in the same direction. In contrast, when the effects of causal variants on the censored trait were in mixed directions, FamKAC outperformed FamBAC and had comparable or higher power than an existing method, RVFam. Our proposed framework has the flexibility to accommodate general nuclear families, and can be used to analyze sequence data for censored traits such as age-at-onset of a complex disease of interest.

## Introduction

Recent advances in next generation sequencing (NGS) have expanded the scope of disease gene mapping from common variants to rare variants. Common genetic variants identified through genome wide association studies (GWASs) often explain only a small proportion of disease heritability. Rare genetic variants, here defined as alleles with a minor allele frequency (MAF) less than 1%–3%, tend to be functional and have stronger effects than common genetic variants, and may account for some of the missing heritability. This transition of disease gene mapping from common variants to rare variants has resulted in active development of new methods that test the association between rare variants and human complex traits. As these variants are rare, single variant association tests, a standard approach for common variants in GWASs, are underpowered unless effect sizes of rare variants or total sample sizes are very large [1, 2]. To overcome this problem, a common strategy is to aggregate rare variants in a gene or region [3–5] in the test statistics. In general, these methods can be classified into two classes: (1) burden tests [1–3], which collapse multiple rare variants into a single burden variable and then test its association with the trait; and (2) variance component or kernel tests (e.g. SKAT [5] and C-alpha [4]), which model marginal effect of each rare variant and then combine into a variance component test. It is known that the burden tests are more powerful than kernel tests when high proportion of rare variants within the region are causal and share effects in the same direction on the trait. Conversely, when a large portion of non-causal rare variants are present or causal rare variants have effects in mixed directions on the trait, kernel tests have advantage over burden tests [5]. To achieve robust power, an optimal test, SKAT-O, was proposed to combine burden and SKAT tests [6].

Although population-based designs are widely used in GWAS, family-based designs have several advantages for rare variants association analyses. First, due to the low minor allele frequencies (MAF) of rare variants, they may not be sampled adequately in the unrelated samples in the population-based designs. On the contrary, family-based designs are likely to increase the chance of sampling causal rare variants because of the shared genetic content within families [7, 8]. Second, by sequencing parents and/or siblings, the chance of observing de novo mutations or rare homozygous genotypes are higher in the family-based designs than in the population-based designs [7]. Lastly, as allele frequencies differ relatively larger in rare variants than in common variants among different geographic regions, populations, or rare variant sites [9, 10], commonly used methods to adjust population stratification such as PCA are likely to fail in population-based designs due to their assumption of a smooth distribution of MAFs [11]. Therefore, family-based designs are appealing because of their known property of being robust to population stratification.

To date, a number of family-based association tests for rare variants have been developed, mostly for qualitative and quantitative traits. Majority of them follow the framework of burden and kernel tests. Here, we further distinguish the family-based association tests for rare variants by whether genotype scores of rare variants are modeled directly (e.g. FBAT-MM [12], KMFAM [13], famBT and famSKAT [14], MONSTER [15]), or decomposed into orthogonal between- and within-family components (*e*.g. [16, 17]). The orthogonal decomposition of genotype scores was initially proposed by Fulker et al. [18], and was later implemented in various family-based association tests for quantitative traits [17, 19, 20]. Abecasis et al. [19] showed analytically that testing the effect of the within-family component of a single common variant is equivalent to testing the additive genetic effect of the variant, and is independent of population stratification. This property was also demonstrated in our recent work on family-based rare variant association tests for quantitative traits [17], in which we showed that our burden and kernel tests built upon the effect of the within-family component remain robust even when simulated datasets consist of subjects from continental subpopulations or closely related subpopulations.

Apart from qualitative and quantitative traits, understanding the genetic basis of censored traits has drawn an increasing interest since the era of linkage studies, such as the effort of mapping genetic modifiers for age-at-onset (AAO) of Alzheimer disease [21, 22]. Here, censored traits refer to time-to-event outcomes. For instance, AAO of a disease is only observed in affected subjects but right censored in unaffected subjects at the last observed age (e.g. age of enrollment into the study or age-at-exam (AAE)). One strategy used in genetic association studies for AAO of a disease was to treat AAO as a quantitative trait [23–25]. However, this strategy is hindered by biased sampling since unaffected subjects, particularly those who may carry genes that affect AAO but have not had disease onset yet, are not included. For this reason, modeling AAOs as a censored trait using survival models can be an attractive alternative. To date, a number of rare variant association methods based on survival models have been developed, mostly for population-based designs [26–28]. In comparison, such methods are rather limited for family-based designs [29, 30]. For family-based designs, the frailty model have advantage over the Cox proportional hazard model since it can incorporate random effects such as the random polygenic effects shared within families. The RVfam R package developed by Chen and Yang [29] can perform rare variants association tests for censored traits in families. Their method utilizes the frailty model to generate test statistics for each individual variant, and then sums over the weighted squares of the test statistics across variants to form a sum of square (SSQ) test [31]. Similar to other methods, RVFam models genotype scores directly. In this paper, we utilized the orthogonal decomposition of genotype scores in the frailty model, and derived the efficient score statistics of the within-family component parameter to construct Family-based Burden (Kernel) Association tests for rare variants with Censored traits, called as FamBAC and FamKAC. The use of the orthogonal decomposition of genotype scores will reduce spurious association findings due to population stratification. In the following sections, we present the analytical derivation of our proposed test statistics, and evaluate their performance through extensive simulation studies.

## Methods

**Fig 1:**
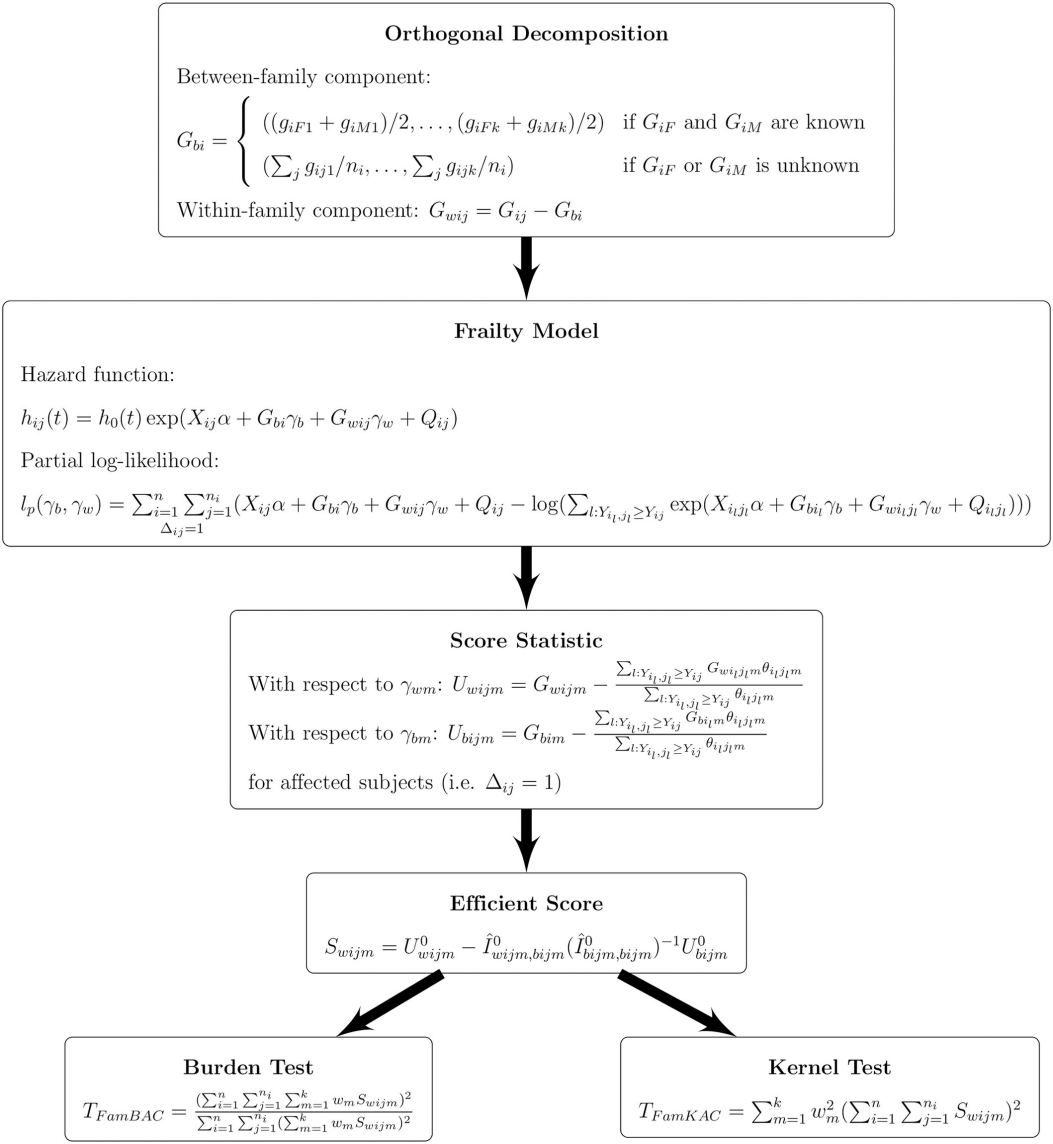
Flowchart for the derivation of FamBAC & FamKAC.

In this section, we describe the general framework of our proposed family-based burden and kernel tests, FamBAC and FamKAC, for detecting the association between rare variants and a censored trait. The main steps of our method derivation are illustrated in a flowchart (Fig 1), including genotype orthogonal decomposition, the frailty model with the between- and within-family genotype components, the score statistics, subject-specific efficient score statistics for each variant, and final burden and kernel test statistics. Further details are described below.

We first introduce the definitions and notations of the orthogonal decomposition of genotype scores for a set of rare variants in general nuclear families, extended from the single variant scenario described in Abecasis et al. [19]. Considering *n* nuclear families and *k* rare variants in a gene or region, we denote a vector of *k* genotype scores as *G*_*ij*_ = (*g*_*ij*1_,…, *g*_*ijk*_) for the *j*^*th*^ (*j* = 1,…, *n*_*i*_) offspring in the *i*^*th*^ (*i* = 1,…, *n*) family (total sample size *N* = ∑_*i*_ *n*_*i*_). For instance, assuming an additive genetic model, the genotype score *g*_*ijm*_ for the *m*^*th*^ variant of the *j*^*th*^ offspring in the *i*^*th*^ family can be coded as 0, 1, or 2 based on the number of minor alleles. For the parental genotype scores in the *i*^*th*^ family, we used the notation of *G*_*iF*_ = (*g*_*iF*1_,…, *g*_*iFk*_) for the father and *G*_*iM*_ = (*g*_*iM*1_,…, *g*_*iMk*_) for the mother, respectively. Following the orthogonal decomposition strategy [18, 19], we composed the genotype scores *G*_*ij*_ into orthogonal between-family component *G*_*bi*_ and within-family component *G*_*wij*_ for the set of *k* rare variants (Fig 1: Orthogonal Decomposition). Both *G*_*bi*_ and *G*_*wij*_ are then incorporated into the frailty model to derive the efficient score statistics for constructing the final test statistics.

### Efficient scores

Let *T* be the event time variable with a finite distribution, such as AAO of a disease, and *C* be the censoring time variable such as AAE. For the *j*^*th*^ offspring (*j* = 1,⋯, *n*_*i*_) in the *i*^*th*^ family (*i* = 1,⋯, *n*), the observed time *Y*_*ij*_ can be denoted as *Y*_*ij*_ = min(*T*_*ij*_,*C*_*ij*_), and the event indicator can be denoted as Δ_*ij*_ = *I*(*T*_*ij*_ ≤ *C*_*ij*_). Taking AAO and AAE of a disease as an example, for the affected subject, the observed time are AAOs (*Y*_*ij*_ = *T*_*ij*_) and the event indicator Δ_*ij*_ = 1; while for the unaffected subject, the observed time are AAEs (*Y*_*ij*_ = *C*_*ij*_) and the event indicator Δ_*ij*_ = 0. Assume that *X*_*ij*_ = (*X*_*ij*1_,…, *X*_*ijp*_) are the *p* covariates and *Q*_*ij*_ is the random polygenic effects from other genes. The hazard function *h*(*t*) at a specific time *t* based on the frailty model is as:

$$\begin{array}{c} h_{ij}\left(t\right)=h_{0}\left(t\right)\exp\left(X_{ij}\alpha +G_{bi}\gamma_{b}+G_{wij}\gamma_{w}+Q_{ij}\right), \end{array}$$ (1)

where *h*_0_(*t*) is the baseline hazard function, and *α* = (*α*_1_,…, *α*_*p*_) is a vector of coefficients for the *p* covariates. *γ*_*b*_ = (*γ*_*b*1_, …, *γ*_*bk*_) and *γ*_*w*_ = (*γ*_*w*1_, …, *γ*_*wk*_) are vectors of parameters for the between- and within-family components of the *k* variants, *G*_*bi*_ and *G*_*wij*_, respectively. The random polygenic effects *Q* follows a normal distribution of *N*(0, *σ*^2^Φ_*i*_ ⊗ *I*), where Φ_*i*_ is the kinship coefficient matrix of family *i*. The partial log-likelihood function can then be written as in Fig 1 (Frailty Model section).

To simplify the presentation, we use parent-offspring trios to illustrate the rest of procedures. The full derivation for general nuclear families with and without parents are described in S1 Appendix. For parent-offspring trios, since there is only one offspring per family, we changed the subject index from *ij* to *i* to simplify the notation. Eq (1) is reduced to:

$$\begin{array}{c} h_{i}\left(t\right)=h_{0}\left(t_{t}\right)\exp\left(X_{i}\alpha +G_{bi}\gamma_{b}+G_{wi}\gamma_{w}+Q_{i}\right)=h_{0}\left(t\right)\theta_{i}. \end{array}$$

Specifically, *Q*_*i*_ ∼ *N*(0, *σ*^2^) and *θ*_*i*_ = exp(*X*_*i*_*α* + *G*_*bi*_*γ*_*b*_ + *G*_*wi*_ *γ*_*w*_ + *Q*_*i*_) with *i* = 1, …, *n* for the offspring in the *i*^*th*^ family. At the variant level, *θ*_*im*_ = exp(*X*_*i*_*α* + *G*_*bim*_*γ*_*bm*_ + *G*_*wim*_*γ*_*wm*_ + *Q*_*i*_) for the *m*^*th*^ variant. The variant-specific partial log-likelihood of the hazard function is then written as:

$$\begin{array}{c} l_{p}\left(\gamma_{bm},\gamma_{wm}\right)=\sum_{i:\Delta_{i}=1}\left(X_{i}\alpha +G_{bim}\gamma_{bm}+G_{wim}\gamma_{wm}+Q_{i}-\log\left(\sum_{l:Y_{l}\geq Y_{i}}\theta_{lm}\right)\right). \end{array}$$ (2)

By taking the first derivative of Eq (2) for each corresponding parameter, we can estimate the fixed effect parameters through maximum partial likelihood estimation on their corresponding score function, and the random effect parameter through penalized partial likelihood approach for frailty model [32, 33] implemented in *‘survival’* R package. Here, we showed the score statistics with respect to *γ*_*wm*_ and *γ*_*bm*_:

$$\begin{array}{c} U_{wm}=\sum_{i:\Delta_{i}=1}U_{wim}=\sum_{i:\Delta_{i}=1}\left(G_{wim}-\frac{\sum_{l:Y_{l}\geq Y_{i}}G_{wlm}\theta_{lm}}{\sum_{l:Y_{l}\geq Y_{i}}\theta_{lm}}\right) \end{array}$$ (3)

and

$$\begin{array}{c} U_{bm}=\sum_{i:\Delta_{i}=1}U_{bim}=\sum_{i:\Delta_{i}=1}\left(G_{bim}-\frac{\sum_{l:Y_{l}\geq Y_{i}}G_{blm}\theta_{lm}}{\sum_{l:Y_{l}\geq Y_{i}}\theta_{lm}}\right), \end{array}$$ (4)

Although the subject-specific score statistics *U*_*wim*_ and *U*_*bim*_ are only defined for affected subjects (i.e. Δ_*i*_ = 1), information from the unaffected subjects is also contained in the score statistics through the summation of subjects whose event or censoring time prior to the event time of an affected subject *i* (i.e. *Y*_*l*_ ≥ *Y*_*i*_ in Eqs (3) and (4)). Evidence for the association between the *k* rare variants and the censored trait can be evaluated through the following hypothesis testing [19]:

$$\begin{array}{l} H_{0}:\gamma_{w}=0\\ H_{a}:\gamma_{w}\neq 0 \end{array}$$

Under the null hypothesis, we can compute the efficient score for each variant by removing the effects of the nuisance parameters (Fig 1). For example, the efficient score of *γ*_*wm*_ for the *m*^*th*^ variant and the *i*^*th*^ trio is as below:

$$\begin{array}{c} S_{wim}=U_{wim}^{0}-{\hat{I}}_{wim,bim}^{0}{\left({\hat{I}}_{bim,bim}^{0}\right)}^{-1}U_{bim}^{0}, \end{array}$$ (5)

where $U_{wim}^{0}$ and $U_{bim}^{0}$ are the score statistics for the within- and between-family components *G*_*wim*_ and *G*_*bim*_, respectively, under the null hypothesis *γ*_*w*_ = 0. ${\hat{I}}_{wim,bim}^{0}$ and ${\hat{I}}_{bim,bim}^{0}$ are components of the observed Fisher information matrix under the null hypothesis (See S2 Appendix).

### Test statistics

The within-family efficient score of each variant from each subject (*S*_*wim*_, Eq (5)), was then used to construct burden and kernel tests. Here, we first introduce several notations in the matrix form. Let *S*_*w*_ be an *n* × *k* matrix of efficient scores with *S*_*w*_[*i*, *m*] = *S*_*wim*_, *W* be a *k* × *k* diagonal weight matrix with *W*[*m*, *m*] = *w*_*m*_ being the weight of the *m*^*th*^ variant. Also let **1**_*n*_ be a vector of *n* 1s and **1**_*k*_ be a vector of *k* 1s. For the burden test, FamBAC collapses efficient scores of multiple rare variants into a single burden variable for each subject to form a test statistics as below:

$$\begin{array}{c} T_{FamBAC}=\frac{{\left(\sum_{i=1}^{n}\sum_{m=1}^{k}w_{m}S_{wim}\right)}^{2}}{\sum_{i=1}^{n}{\left(\sum_{m=1}^{k}w_{m}S_{wim}\right)}^{2}}=\frac{{\left(S_{w}^{T}1_{n}\right)}^{T}W1_{k}1_{k}^{T}W\left(S_{w}^{T}1_{n}\right)}{{\left(S_{w}W1_{k}\right)}^{T}\left(S_{w}W1_{k}\right)}. \end{array}$$ (6)

We can prove that all elements of *S*_*w*_ are asymptotically distributed as a normal distribution with mean **0** under the null hypothesis. It is also reasonable to assume that under the null hypothesis the efficient scores of the offsprings in each trio are independently identically distributed. Then, according to the central limit theorem, *T*_*FamBAC*_ is asymptotically distributed as *χ*^2^ distribution with degree of freedom 1.

For the kernel test, FamKAC can be written as a quadratic statistic which sums over the marginal effects of individual rare variants as below:

$$\begin{array}{c} T_{FamKAC}=\sum_{m=1}^{k}w_{m}^{2}{\left(\sum_{i=1}^{n}S_{wim}\right)}^{2}={\left(S_{w}^{T}1_{n}\right)}^{T}W^{2}\left(S_{w}^{T}1_{n}\right). \end{array}$$ (7)

Under the null hypothesis, $S_{w}^{T}1_{n}=(\sum_{i=1}^{n}S_{wi1},\ldots ,\sum_{i=1}^{n}S_{wik}{)}^{T}$ is asymptotically distributed as a multivariate normal distribution with mean 0 and covariance matrix *n* × *cov*(*S*_*w*1_, *S*_*w*2_, …, *S*_*wk*_), where *S*_*w*1_, *S*_*w*2_, …, *S*_*wk*_ are the efficient scores of the *k* variants from *S*_*w*_. This quadratic form is shown to follow a mixture of *χ*^2^ distribution as:

$$\begin{array}{c} T_{FamKAC}∼\sum_{m=1}^{k}\lambda_{m}\chi^{2}\left(1\right), \end{array}$$

where λ_1_, …, λ_*k*_ are the eigenvalues of *V* = *nW*^2^*cov*(*S*_*w*1_, *S*_*w*2_, …, *S*_*wk*_). For computing efficiency, Davies exact method [34] can be used to approximate the mixture of *χ*^2^ distribution to obtain *p*-values. Alternatively, one can also perform permutation tests to obtain empirical *p*-values.

It has been shown that decreasing the weight of non-causal variants and increasing the weight of causal variants can yield improved power. To improve the performance of the tests in detecting effects of very low frequency variants, it will require choosing a suitable variant weighting scheme that would up-weight the variants with lower frequencies. Let *w*_1_, …, *w*_*k*_ be the weights of the *k* variants, which are pre-specified without using the outcome and reflects the relative contribution of the variants to the test statistic. There have been several weighting schemes proposed to date [2, 5, 17]. Here, we focus on two commonly used variant weight functions: the Madsen and Browning weight $w_{p}=1/\sqrt{p(1-p)}$ [2] and the Beta weight *w*_*B*_ = *Beta*(*p*; 1, 25) [5], where *p* refers to the MAF of a given variant.

### Simulation studies

To evaluate the validity and performance of our proposed methods FamBAC and FamKAC, we conducted a series of simulation studies of parent-offspring trios, and assessed Type I errors and statistical power under various parameter settings. We designed our simulation of sequence data based on the exome of a gene region, which is similar to the process described in Jiang et al. [17]. That is, assuming a gene with a size of 30 kb, we first generated a pool of 20,000 haplotypes of size 30 kb using COSI [35] (https://personal.broadinstitute.org/sfs/cosi/), with parameters that mimicked linkage disequilibrium and allele frequency distributions observed in European population. We then divided this 30 kb gene into 100 segments of size 300 base pairs to represent subregions of the gene (*e*.g. introns, exons). Assuming 10 exons are within a gene, we randomly select 10 segments from the 100 segments to form a haplotype size of 3 kb to represent the exome captured in whole exome sequencing. This 3 kb region was subsequently fixed for all replicates generated by simulation. Using the pool of 20,000 haplotypes of size 3 kb, we generated genotype data for a population of 1,000,000 parent-offspring trios. Specifically, for each parent-offspring trio, we first randomly sampled two haplotypes without replacement from the 20,000 haplotype pool to form genotype data for each parent, and then randomly sampled one haplotype from each parent to generate genotype data for the offspring. The variants considered to be rare were those with population MAF from COSI less than 0.01. Consequently, 69 rare variants were found within the selected 3 kb region.

To simulate AAO of a disease as a censored trait, we assumed that every subject will eventually encounter the disease given sufficient follow-up time. Therefore, in order to create the censoring scenario, we generated AAO and AAE separately for each offspring. When *AAO* ≤ *AAE*, the subject was designated as affected with event time at AAO and event indicator Δ = 1; when *AAO* > *AAE*, the subject was designated as unaffected with censoring time at AAE and Δ = 0. To evaluate type I error rates, the AAO data were generated under the null hypothesis, where rare variants within the 3 kb region do not affect the AAO distribution. Thus, we drew random samples of AAO from a normal distribution *N*(*μ*_*AAO*_, *V*_*AAO*_), where *μ*_*AAO*_ and *V*_*AAO*_ were the pre-specified mean and variance of AAO. Similarly, AAE was randomly sampled from another normal distribution *N*(*μ*_*AAE*_, *V*_*AAE*_) where *μ*_*AAE*_ and *V*_*AAE*_ were the pre-specified mean and variance of AAE. For simplicity, we assumed the variance of AAO and AAE were the same (*V*_*AAE*_ = *V*_*AAO*_ = 100). To ensure that the AAO data were generated within a reasonable life span (e.g. within 100 years of age), we assumed that the mean AAO (*μ*_*AAO*_) was at the age of 45. The mean AAE (*μ*_*AAE*_) was determined to be at an age that can theoretically lead to a given censoring rate. For example, to reach 10% censoring rate, *μ*_*AAE*_ was estimated at 26.88. For each parameter setting, we generated 10,000 replicates of 2,000 trios to estimate type I error rate.

To evaluate the power of proposed tests, AAO data were generated under the alternative hypothesis, where rare variants within the 3 kb region contribute to the AAO distribution. We assumed that the total AAO variance (*V*_*AAO*_) was contributed by all causal rare variants within the 3 kb region (*V*_*g*_), polygenes (*V*_*p*_), and residual environmental factors (*V*_*e*_). We fixed the total AAO variance *V*_*AAO*_ at 100 and the residual variance *V*_*e*_ at 75. This led to various combinations of *V*_*g*_ and *V*_*p*_ for the remaining variance (*V*_*g*_ + *V*_*p*_ = 25). We set different proportions of causal rare variants (*P*_*cv*_) to obtain the number of causal rare variants (*k*_*cv*_) in the region. We randomly selected *k*_*cv*_ causal rare variants from all rare variants within the 3 kb region. Assuming all causal rare variants contributed equally to AAO, the genetic variance contributed by each causal rare variant was obtained from $V_{cv}=\frac{V_{g}}{k_{cv}}$. We can then derive the additive genetic effect of each causal variant by $a_{m}=\sqrt{\frac{V_{cv}}{p_{m}(1-p_{m})}}$, where *p*_*m*_ was the population MAF for the *m*^*th*^ variant from COSI. The AAO data of the offspring in the *i*^*th*^ trio was then generated from the following model, $AAO_{i}=\mu_{AAO}+\sum_{m=1}^{k_{cv}}a_{m}(g_{im}-1)+P+E$, where *μ*_*AAO*_ was the overall AAO mean fixed at 45, *g*_*im*_ was the genotype score of the offspring in the *i*^*th*^ trio at the *m*^*th*^ variant, and *P* and *E* were the random polygenic and environmental effects, randomly drawn from *N*(0, *V*_*p*_) and *N*(0, *V*_*e*_), respectively. Similar to type I error simulations, AAE was randomly sampled from *N*(*μ*_*AAE*_,*V*_*AAE*_), where *V*_*AAE*_ was fixed at 100 and *μ*_*AAE*_ was determined at the age to theoretically achieve a given censoring rate. Table 1 summarizes the parameter settings used in the simulation studies.

**Table 1:** Parameters used in the simulations.

Size of Haplotype Pool	20,000
Size of Region	3 kb
Minor Allele Frequencies (MAF) to Consider Rare	< 0.01
Number of Rare Variants within the 3 kb Region	69
Censoring Rate	0.1, 0.2, 0.3, 0.5
Total Variance of Age-at-Onset (AAO) (*V*_*AAO*_) and Age-at-Exam (AAE) (*V*_*AAE*_)	100
Variance of AAO Contributed by All Causal Rare Variants (*V*_*g*_)	5, 10, 15, 20
Variance of AAO Contributed by Polygenes (*V*_*p*_)	20, 15, 10, 5
Proportion of Rare Variants to be Causal (*P*_*cv*_)	0.1, 0.3, 0.5, 0.7, 0.9, 1
Number of Parent-Offspring Trios	1,000, 2,000, 3,000, 4,000
Number of Replicates	1,000, 10,000

Our simulation studies covered two scenarios regarding to the directions of effects of the *k*_*cv*_ causal rare variants within the 3 kb region on AAO. First, we simulated one scenario in which all *k*_*cv*_ causal rare variants had negative effects on AAO. In other words, all causal rare variants would lead to earlier AAO (deleterious variants). By fixing all other parameters, we compared statistical power of the proposed methods FamBAC and FamKAC for different censoring rates, variance of AAO contributed by all causal rare variants (*V*_*g*_), proportions of rare variants to be causal (*P*_*cv*_), and number of trios, respectively. Second, we simulated another scenario in which the *k*_*cv*_ causal rare variants had effects in mixed directions on AAO. Within the *k*_*cv*_ causal rare variants, we randomly selected a proportion of causal rare variants with positive effects on AAO, which would lead to later AAO (positve directon), and the rest of causal rare variants with negative effects on AAO (negative direction). We compared our proposed methods FamBAC and FamKAC with RVFam [29] for three different postive/negative direction setting: 0/100, 20/80, and 50/50. For all simulations of power, we generated 1, 000 replicates of 2, 000 trios, except when we compared different number of trios.

## Results

### Type I error

To estimate type I error rates, we simulated 10, 000 replicates of 2, 000 trios for three levels of censoring rates: 0.1, 0.2, and 0.3). We investigated five rare variant weighting schemes for FamBAC and FamKAC, including (1) *w*_0_ = 1 no weight; (2) $w_{p}=1/\sqrt{p(1-p)}$ with population MAF *p* from COSI; (3) $w_{\hat{p}}=1/\sqrt{\hat{p}(1-\hat{p})}$ with sample MAF $\hat{p}$ estimated from *G*_*b*_ ($\hat{p}=\sum_{i=1}^{n}G_{bi}/2n$); (4) *w*_*B*_ = *Beta*(*p*; 1, 25) the density function of *Beta*(1, 25) distribution at population MAF *p*; and (5) $w_{B}=Beta(\hat{p};1,25)$ the density function of *Beta*(1, 25 distribution at sample MAF $\hat{p}$. Davies’ approximation method was used to estimate *p*-values of FamKAC for computational efficiency.

Table 2 summarizes the type I error rates of FamBAC and FamKAC for different combinations of censoring rates and variant weighting schemes. The type I errors of FamBAC were generally close to 0.05 for all censoring rates and weighting schemes. The variant weights of *w*_0_, *w*_*B*_, and $w_{\hat{B}}$ led to better controlled type I errors (ranging 0.0469 − 0.0527) than the variant weights of *w*_*p*_ and $w_{\hat{p}}$ (ranging 0.0371 − 0.0465). On the other hand, FamKAC generally had deflated type I errors (maximum at 0.0376), especially when Madsen and Browning weight *w*_*p*_ and $w_{\hat{p}}$ were used. To further examine the type I error of FamKAC, we conducted one set of simulation based on permutation tests for the setting of censoring rate at 30% with rare variants weighted by $w_{\hat{B}}$. We performed 1, 000 permutations to obtain an empirical *p*-value for each replicate, and computed type I error based on 1, 000 replicates, a reduced number of replicates due to the computational burden. The type I error was improved from 0.0330 to 0.0424, which was encouraging to re-assure the validity of FamKAC. Finally, our simulation showed that population MAF *p* and sample MAF $\hat{p}$ did not differ type I error much when the same variant weight function was used. Based on the above observations on different variant weighting schemes, we only applied weights *w*_0_ and $w_{\hat{B}}$ to simulate power.

**Table 2:** Type I error rates of FamKAC and FamBAC for different censoring rates.

	Censoring Rate	*w*_0_ = 1	$w_{p}=1/\sqrt{p(1-p)}$ ^†^	$w_{\hat{p}}=1/\sqrt{\hat{p}(1-\hat{p})}$ ^‡^	*w*_*B*_ = *Beta*(*p*; 1, 25)	$w_{\hat{B}}=Beta(\hat{p};1,25)$
FamBAC	0.1	0.0498	0.0428	0.0432	0.0483	0.0482
	0.2	0.0485	0.0414	0.0431	0.0469	0.0471
	0.3	0.0477	0.0432	0.0465	0.0479	0.0480
	0.5	0.0524	0.0371	0.0437	0.0527	0.0527
FamKAC	0.1	0.0376	0.0062	0.0103	0.0359	0.0358
	0.2	0.0367	0.0042	0.0084	0.0354	0.0359
	0.3	0.0353	0.0048	0.0091	0.0338	0.0330
	0.5	0.0367	0.0039	0.0065	0.036	0.0359

^†^ *p* is the population minor allele frequencies (MAF) from COSI;

^‡^ $\hat{p}$ is the sample MAF estimated from the between-family component *G*_*b*_; All p-values were estimated based on 10, 000 replicates of 2, 000 trios.

### Power

To evaluate statistical power, 1, 000 replicates were simulated for each parameter setting. The parameters considered in the simulation studies included censoring rate, variance of AAO contributed by all causal rare variants *V*_*g*_, number of trios, proportion of rare variants to be causal (*P*_*cv*_) (Table 1). We chose 30% censoring rate, *V*_*g*_ = 5, *n* = 2, 000 trios, and *P*_*cv*_ = 30% as the default parameter setting. For each simulation, we varied one parameter at a time and fixed the other parameters at the default values. For example, to evaluate the effect of censoring rate, we simulated censoring rates at 10%, 20%, 30%, and 50%, respectively, with other parameters fixed at *V*_*g*_ = 5, *n* = 2, 000, and *P*_*cv*_ = 30%.

**Fig 2:**
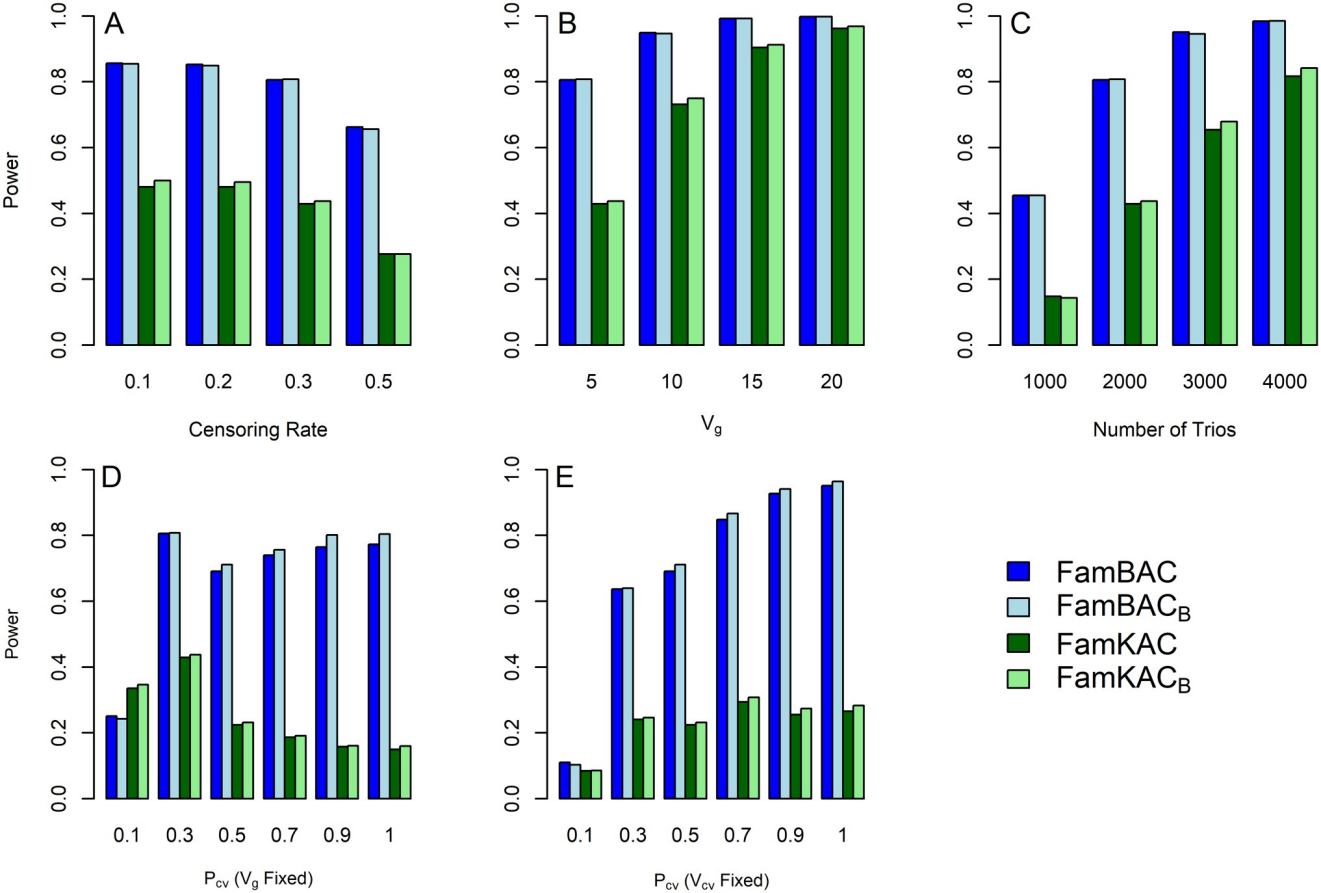
Power comparison for FamBAC and FamKAC when causal variants are in the same direction of effects on age-at-onset (AAO) trait. Power was estimated based on 1,000 replicates under the following parameter settings: (A) censoring rate: 0.1, 0.2, 0.3, and 0.5; (B) *V*_*g*_: 5, 10, 15, and 20; (C) number of trios: 1, 000, 2, 000, 3, 000, and 4, 000; (D) *P*_*cv*_: 9.1, 0.3, 0.5, 0.7, and 1 under *V*_*g*_ fixed at 5; (E) *P*_*cv*_: 0.1, 0.3, 0.5, 0.7, and 1 under fixed *V*_*cv*_ for all causal variants, where *V*_*cv*_ is derived based on *V*_*g*_ = 5 and 50% of rare variants being causal. The default parameter setting for all simulations is 30% censoring rate, *V*_*g*_ = 5, 2, 000 trios, and *P*_*cv*_ = 30%. Method notations are the following: FamBAC: FamBAC with no variant weight *w*_0_ = 1; FamBAC_*B*_: FamBAC with variant weight $w_{\hat{B}}=Beta(\hat{p};1,25)$ where $\hat{p}$ is the sample minor allele frequency (MAF) estimated from the between-family component *G*_*b*_; FamKAC: FamKAC with *w*_0_; FamKAC_*B*_: FamKAC with variant weight $w_{\hat{B}}$.

First, we simulated the scenario in which all causal rare variants had the same direction of negative effects on AAO. Fig 2 depicts the statistical power of FamBAC and FamKAC under various parameter settings. As expected, both burden and kernel tests had decreased power when censoring rates increased (Fig 2A), and increased power when *V*_*g*_ or number of trios increased (Fig 2B and 2C). When all causal rare variants had negative effects on AAO, the burden tests consistently outperformed the kernel tests. Interestingly, there is less consistent power pattern under different *P*_*cv*_, proportions of causal rare variants. Under fixed *V*_*g*_, the 10% causal rare variant scenario shows the lowest statistical power (24.2%) for FamBAC while other proportions (*P*_*cv*_ = 30% − 100%) have similar power, ranging from 71.1% to 80.8% (Fig 2D). On the contrary, for kernel test FamKAC, *P*_*cv*_ has a negative effect on the power, that is, FamKAC tends to have decreased power as *P*_*cv*_ increased. This may be due to the fact that under fixed *V*_*g*_, the AAO variance contributed by each causal rare variant ($V_{cv}=\frac{V_{g}}{k_{cv}}$) decreased as the number of causal rare variants *k*_*cv*_ increased. Therefore, we conducted another set of simulations where *V*_*cv*_ was fixed for all causal rare variants in the region for all different *P*_*cv*_ scenarios. We set the *V*_*cv*_ at the measure derived from the scenario of *V*_*g*_ = 5 and *P*_*cv*_ = 50%. This set of simulations showed that both FamBAC and FamKAC had the lowest power when 10% of rare variants are causal. However, under the scenarios of *P*_*cv*_ ≥ 30%, FamBAC showed increasing power as *P*_*cv*_ increased, but FamKAC showed similar power across different *P*_*cv*_ (Fig 2E).

**Fig 3:**
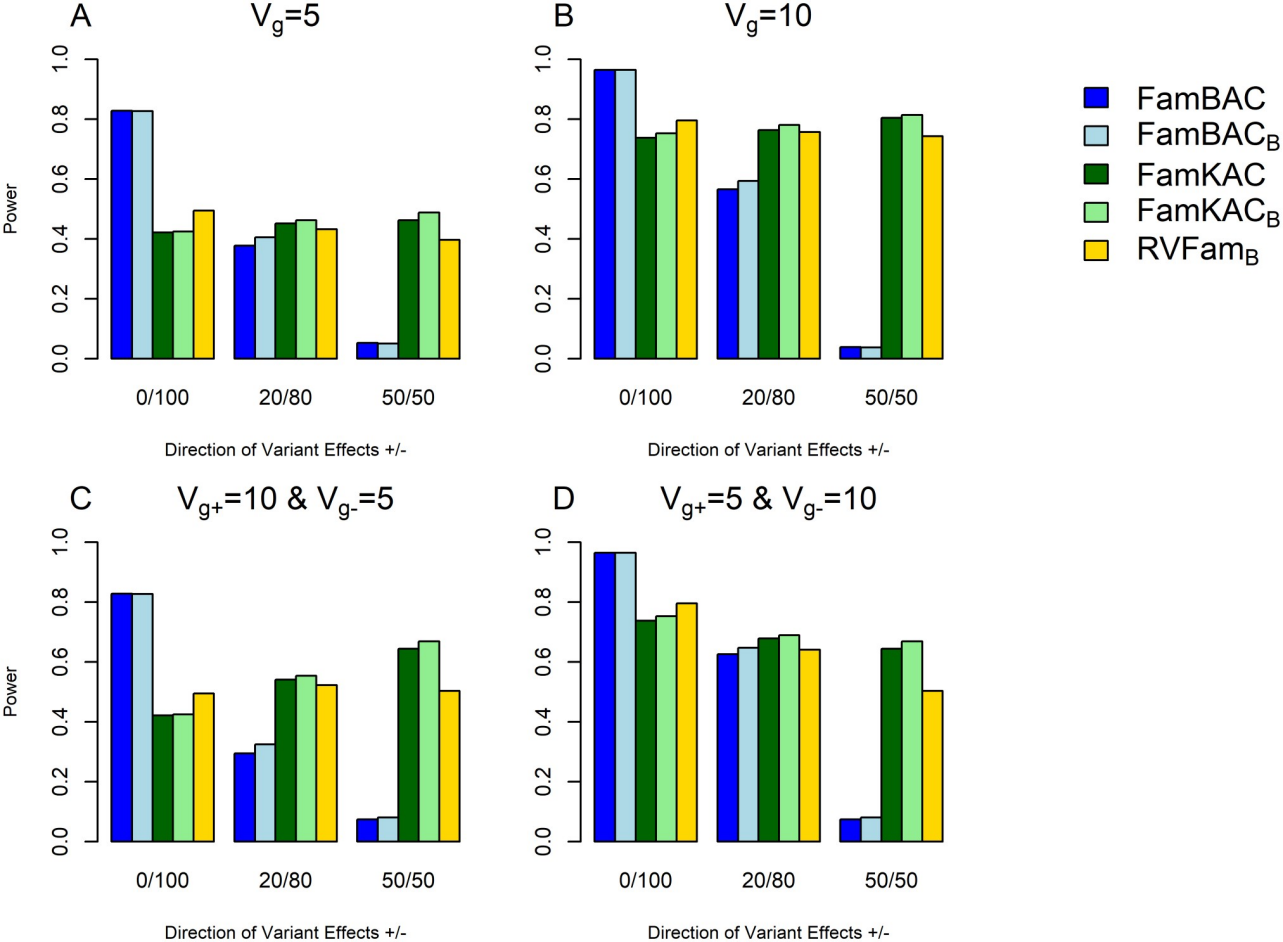
Power comparison for FamBAC, FamKAC, and RVFam for effects of causal rare variants in mixed directions. Simulations were based on 1, 000 replicates with causal rare variants having effects in mixed directions (+/-: 0/100, 20/80, and 50/50) on age-at-onset (AAO) for different *V*_*g*_: (A) *V*_*g*_ = 5; (B) *V*_*g*_ = 10; (C) *V*_*g*+_ = 10 for causal rare variants with positive effects and *V*_*g*−_ = 5 for causal rare variants with negative effects; and (D) *V*_*g*+_ = 5 for causal rare variants with positive effects and *V*_*g*−_ = 10 for causal rare variants with negative effects. The other parameters were fixed at 30% censoring rate, 2, 000 trios, and 30% causal rare variants. Method notations are as the following: FamBAC: FamBAC with no variant weight *w*_0_ = 1; FamBAC_*B*_: FamBAC with variant weight $w_{\hat{B}}=Beta(\hat{p};1,25)$ where $\hat{p}$ is the sample minor allele frequency (MAF) estimated from the between-family component *G*_*b*_; FamKAC: FamKAC with *w*_0_; FamKAC_*B*_: FamKAC with variant weight $w_{\hat{B}}$; and RVFam_*B*_: RVFam with variant weight $w_{\hat{B}}$.

Next, we simulated the scenario in which the causal rare variants had effects in mixed directions on AAO. We set three different proportions of causal rare variants in positive/negative effects on AAO as 0/100, 20/80, and 50/50. For instance, 20/80 means that 20% of causal rare variants have positive effects on AAO while 80% of causal rare variants have negative effects on AAO. In addition to FamBAC and FamKAC, we added RVFam test [29] for comparison. Fig 3 depicts the statistical power for these three tests under different positive/negative direction effects for four *V*_*g*_ settings (5, 10, and two combinations). Similarly, the other parameters were fixed at the default values of 30% censoring rate, *n* = 2, 000 trios, and 30% of causal rare variants. We showed that FamBAC has decreasing power as the ratio of causal rare variants with different directions of effects increases (*e*.g. 20/80 and 50/50) for all *V*_*g*_ settings. FamKAC has fairly constant power across different positive/negative direction settings under a fixed *V*_*g*_ = 5 or *V*_*g*_ = 10 (Fig 3A and 3B) but showing decreasing or increasing pattern when different *V*_*g*_ was given for a specific direction, for instance, *V*_*g*+_ = 10 for causal rare variants with positive effects on AAO and *V*_*g*−_ = 5 for causal rare variants with negative effects on AAO, or vise versa (Fig 3C and 3D). RVFam showed slightly higher power than FamKAC when all variants are in the same direction (0/100 scenario) even though its power is still lower than FamBAC. Under the mixed direction effect scenarios (20/80 and 50/50), FamKAC had slightly higher power than RVFam across all *V*_*g*_ settings. This power difference between FamKAC and RVFam is more apparent under the scenario of 50/50 mixed direction of effects on AAO (*e*.g. 0.67 vs. 0.50 for 50/50 in Fig 3C and 3D).

Two variant weighting schemes were used in the simulation studies to assess the statistical power of FamBAC and FamKAC. While the power difference between Beta variant weight ($w_{\hat{B}}$) and no given weight (*w*_0_) is small for all simulation studies (Figs 2 and 3), Beta variant weight tends to have slightly higher power than those with *w*_0_, particularly for FamKAC.

## Discussion

We propose a set of family-based burden and kernel tests, referring to as FamBAC and FamKAC, for identifying association between rare variants and time-to-event outcomes. Both methods were built upon the efficient scores of parameter for the within-family genotype component derived from the frailty model. Our proposed methods utilized several known statistical properties. First, the orthogonal decomposition of offspring genotype scores into between- and within-family components is applicable to nuclear families of any sizes with and without parental genotype data. It is known that testing the effect of within-family component on the trait is robust to the potential population stratification [19]. Second, the frailty model is one of the survival models that can incorporate random effects. Therefore, this model will allow us to account for the random polygenic effects on the censored traits. Third, since the score statistics is derived under the null hypothesis (*γ*_*w*_ = 0), it is known to be more stable and computational efficient than the likelihood ratio test. Moreover, using efficient score statistics instead of score statistics has the advantage of removing the effects of nuisance parameters. Finally, our proposed tests have the flexibility to use different variant weighting schemes of one’s choice. Like other burden and kernel tests, p-values can be obtained analytically based on the corresponding distribution of the test statistics, $\chi_{\left(1\right)}^{2}$ distribution for FamBAC and a mixture of *χ*^2^ distributions for FamKAC. Alternatively, permutation tests can be employed to increase the accuracy of p-values, particularly for FamKAC.

The simulation studies under the null hypothesis showed that type I error was well controlled for FamBAC but deflated for FamKAC under different simulation settings. However, the validity of FamKAC is still supported by permutation tests. This deflation is likely due to the fact that we used Davies’ approximation method [34] to generate p-values for a mixture of *χ*^2^ distributions for FamKAC. This deflated type I error may also explain why FamKAC had lower power than we would expect in simulation studies. Since Davies’ approximation method can compute p-values efficiently, one may want to screen all genes using Davies’ method for FamKAC first, and then conduct permutation tests on selected genes meeting a conservative threshold (*e*.g. Davies *p* ≤ 0.1) for confirmation.

For the performance of FamBAC and FamKAC, their statistical power is generally aligned with what we anticipated for burden and kernel types of methods. That is, FamBAC performed better than FamKAC when majority of causal rare variants have same direction of effects, and conversely FamKAC outperformed FamBAC when causal rare variants have effects in mixed directions. This is anticipated as the burden tests directly collapse all rare variants without distinguishing the sign of their effect. On the other hand, since FamKAC is a variance component test using the second central moment statistics, it captures the absolute effects of all rare variants. Therefore, FamKAC is robust to the scenario of variants with mixed direction effects to the trait. Considering rare variants are most likely new mutations, it is not realistic to assume that all of causal rare variants affect the trait in the same direction. Since the direction of the variant effect is unknown and can be varied by genes, in practice, both tests should be performed. With the complementary property of both tests, it is also possible to implement a combined test of FamBAC and FamKAC using the similar strategy proposed in SKAT-O [6] in the future, but more evaluations are needed.

The genetic variance contributed by all causal rare variants (*V*_*g*_) and individual causal rare variant (*V*_*cv*_) are correlated with the proportion of causal rare variants (*P*_*cv*_) in the gene. Under fixed *V*_*g*_, the *V*_*cv*_ decreases as *P*_*cv*_ increases, while under fixed *V*_*cv*_ for all causal rare variants, *V*_*g*_ increases as *P*_*cv*_ increases. In practice, these three parameters are unknown for a given gene. However, it is probably reasonable to assume that *P*_*cv*_ is lower in the full gene sequence from whole genome sequencing but larger in the exome of the gene [5]. For this reason, we evaluated the power of our methods for the full spectrum of *P*_*cv*_ from 10% to 100% in related to *V*_*g*_ and *V*_*cv*_. Under the 10% causal variant scenario, the power of both tests were generally lower than other proportions regardless of fixing *V*_*g*_ or *V*_*cv*_ (Fig 2D and 2E). This may be caused by the fact that majority of rare variants included in the test (90% of them in the gene) were non-informative, which may override the signals from causal variants in the test statistics. For *P*_*cv*_ ≥ 30%, when *V*_*g*_ is fixed, FamBAC (burden test) maintained similar power across different *P*_*cv*_, while FamKAC (kernel test) showed decreasing power as *P*_*cv*_ increased (Fig 2D). Although one may argue that the power of FamBAC seems to peak at 30% causal rare variant scenario (Fig 2D), the difference is actually small (*e*.g. 80.09% power for *P*_*cv*_ = 30% vs. 79.9% for *P*_*cv*_ = 90%). The above power pattern for the scenarios with *P*_*cv*_ ≥ 30% may be explained by how burden and kernel tests handle the effects of rare variants. When *V*_*g*_ is fixed, the higher *P*_*cv*_ will result in lower *V*_*cv*_. Since burden tests collapse the effects of all rare variants, *V*_*g*_ is preserved within each subject regardless of the different quantity of *V*_*cv*_. On the other hand, kernel tests compute variance components for each variant prior to summing over variants. Therefore, the smaller *V*_*cv*_ may lead to smaller test statistics and then lower power. In contrast, when *V*_*cv*_ is fixed for all causal rare variants, the increase of *P*_*cv*_ is equivalent to the increase of *V*_*g*_. As expected, burden test (FamBAC) shows increasing power as *P*_*cv*_ increased. Conversely, under fixed *V*_*cv*_ for all rare variants, the variance component that capture the variation among rare variants is likely small between different *P*_*cv*_, which may explain the relatively consistent power observed in FamKAC. While the proportion of causal rare variants (*P*_*cv*_) is a key factor to influence the power of both tests, we conclude that FamBAC is more sensitive to the total genetic variance (*V*_*g*_), and FamKAC is more sensitive to the effect size of individual variant (*V*_*cv*_).

Similar to FamKAC, the existing method RVFam had lower power than FamBAC when all causal rare variants have the same direction of effect to the trait, but higher power than FamBAC under the mixed direction of the effect (Fig 3). This is somewhat anticipated as RVFam is based on sum of square statistics that also capture the absolute effect size of each rare variant. However, minor performance differences were still observed between FamKAC and RVFam. We found that RVFam performed better than FamKAC when all causal rare variants have the same direction of effects on the trait. However, in all scenarios of mixed direction effects, FamKAC showed consistent higher power than RVFam, particularly when it is under 50/50 mixed direction of effects on the trait. While both methods are generally comparable, FamKAC should have more chances to outperform RVFam. Although both methods utilized the frailty model, the derivation process is different between them, specifically, on how the genotype scores were treated in the model and how the test statistics were constructed. Further, since FamKAC incorporates the orthogonal decomposition of genotype scores, it should be more robust than RVFam for data with population stratification.

Cox proportional hazard (PH) model is the most commonly used method for analyzing survival outcomes. In this study, we chose to use the frailty model instead of Cox PH model due to the consideration of the random polygenic effects shared within families. It is important to include the ploygenic effects as a random effect in the model because a complex disease is mostly affected by multiple genes rather than a single gene. For this reason, the frailty model, a generalization of the Cox PH model, fits better than Cox PH for family-based design. Regarding the applications of our proposed FamBAC and FamKAC, it is worthwhile to note the differences between our proposed methods for censored traits and commonly used methods for quantitative traits. Using AAO of a disease as an example, when AAO is treated as a quantitative trait, it is equivalent to a case-only design where only affected subjects with AAO are included. With the biased sampling on affected only, the distribution of AAO is likely to be skewed, and large sample size from affected subjects will be required to achieve sufficient power. The quantitative trait methods focus more on the amount of AAO changes if one carries the minor alleles of rare variants in the gene. On the other hand, our proposed tests that treat AAO as a censored trait can include the observed event time (AAO) from affected subjects and censoring time (AAE) from unaffected subjects. Our results focus on the relative risk (hazard ratio) of developing the disease at any age between the risk allele carriers and the non-carriers for the gene of interest.

Some limitations are existed in this study. First, we used an arbitrary sequence length in our simulation studies. Our intention was to mimic the exonic region of a gene by generating 10 exons made up from 10 equal sequence length (300 bp). In reality, the number of exons and exon length may vary in/between genes. However, these arbitrary settings should not impact the pattern of simulation results much because the proposed method are gene-based test that aggregate all rare variant effects in the gene. The total genetic variance (*V*_*g*_) and the proportion of causal rare variants (*P*_*cv*_) in the region have more impact on the statistical power, which have been captured in our simulation studies. Since the full 30 kb haplotype sequences were simulated based on the European genome, they should have captured the similar LD structure and allele frequencies in the European genome. Similarly, the exonic region we assembled should still capture the LD structure within each exon as well as across exons. Overall, our simulated sequence data should have sufficient representation of the sequence structure in the European genome for method evaluation. Second, we assume that all causal rare variants have equal effect size, which is unlikely in the real data. If *V*_*cv*_ varies across rare variants in the gene, this should not affect the performance of FamBAC since the *V*_*g*_ (sum of *V*_*cv*_ effects in the gene) is more relevant to FamBAC. Based on our simulation results shown in Fig 2D and 2E, under a fixed *V*_*g*_, we would anticipate FamKAC shown higher power for the scenario of higher proportion of variants with higher *V*_*cv*_ than the scenario of higher proportion of variants with lower *V*_*cv*_. Third, our proposed methods focus on testing rare variants within the region of interest, not including common variants. Common disease common variants (CDCV) and common disease rare variants (CDRV) [36] are two well-known hypotheses for genetic contributions to human complex diseases. CDCV argues that the common variants contribute to disease susceptibility with low disease penetrance, while CDRV argues that rare variants have relatively high penetrance. Clearly, identifying common and rare variants are equally important to explain the genetic heritability of the disease. In general, single variant tests are applied to common variants while gene-based methods are used for rare variants. There are also gene-based methods proposed to test common and rare variants together by using different variant weights [37] but have not been used widely in the NGS data analysis. Our methods can contribute to the area of identifying rare variants for trait with censoring. Finally, we are still in the process of developing an R package for the proposed methods for data analysis. The simulation programs were developed in R and ran under the UNIX system for all replicates. We will release our R package to public once it is ready.

We have proposed two rare variant association tests, FamBAC and FamKAC, for censored traits under family-design. Although we used parent-offspring trios throughout most part of this paper to describe our methods, as shown in the supporting information section, both methods can be applied to nuclear families of any sizes with and without parental genotypes. We have also shown the complementary property of FamBAC and FamKAC through simulation studies. In conclusion, as the genetic basis of AAO for human complex diseases remains an important area of research, our proposed methods can facilitate the analysis of family-based next generation sequencing data to identify gene associated with disease AAO.

## Supporting information

S1 Appendix FamBAC and FamKAC for general nuclear families. (PDF)

S2 Appendix Observed Fisher information matrix for parent-offspring trios. (PDF)

S3 Appendix Parameter settings for COSI in simulation studies. (PDF)
