# Supplementary material for: Family-based association tests for rare variants with censored traits
Source: PLoS One. 2019 Jan 25;14(1):e0210870. doi: 10.1371/journal.pone.0210870 (PMC6347269; doi:10.1371/journal.pone.0210870)
Supplement: S1 Appendix — (PDF) [file pone.0210870.s001.pdf]

S1 Appendix: FamBAC and FamKAC for general nuclear families

For general nuclear families with or without parental genotypes, burden and kernel tests, FamBAC and FamKAC, can be constructed using the orthogonal genotype decomposition in the similar way as described in the methods section for parent-offspring trios.

Considering the mth variant and $\theta_{ijm} = \exp(X_{ij}\alpha + G_{bim}\gamma_{bm} + G_{wijm}\gamma_{wm} + Q_{ij})$, we can write out the partial log-likelihood of the hazard function in the frailty model as:

$$l_p(\gamma_{bm}, \gamma_{wm}) = \sum_{i=1}^{n} \sum_{\substack{j=1 \\ \Delta_{ij}=1}}^{n_i} (G_{bim}\gamma_{bm} + G_{wijm}\gamma_{wm} - \log(\sum_{l:Y_{i_l,j_l} \geq Y_{ij}} \theta_{i_l j_l m})),$$

for n_i siblings in the ith family, where $i = 1, \cdots, n$. Similarly, by taking the first derivative for each corresponding parameter, we can estimate the fixed effect parameters through maximum partial likelihood estimation on their corresponding score function, and the random effect parameter through penalized partial likelihood approach for frailty model [1, 2] implemented in *'survival'* R package. The two main score statistics with respect to γ_{wm} and γ_{bm} are shown as below:

$$U_{wm} = \sum_{i=1}^{n} \sum_{\substack{j=1 \\ \Delta_{ij}=1}}^{n_i} U_{wijm} = \sum_{i=1}^{n} \sum_{\substack{j=1 \\ \Delta_{ij}=1}}^{n_i} (G_{wijm} - \frac{\sum_{l:Y_{i_l,j_l} \geq Y_{ij}} G_{wi_l j_l m}\theta_{i_l j_l m}}{\sum_{l:Y_{i_l,j_l} \geq Y_{ij}} \theta_{i_l j_l m}})$$

and

$$U_{bm} = \sum_{i=1}^{n} \sum_{\substack{j=1 \\ \Delta_{ij}=1}}^{n_i} U_{bijm} = \sum_{i=1}^{n} \sum_{\substack{j=1 \\ \Delta_{ij}=1}}^{n_i} (G_{bim} - \frac{\sum_{l:Y_{i_l,j_l} \geq Y_{ij}} G_{bi_l m}\theta_{i_l j_l m}}{\sum_{l:Y_{i_l,j_l} \geq Y_{ij}} \theta_{i_l j_l m}}).$$

Similar to parent-offspring trios, the observed Fisher information matrix can be written as:

$$\hat{I}_p = \begin{pmatrix} \hat{I}_{bm,bm} & \hat{I}_{bm,wm} \\ \hat{I}_{wm,bm} & \hat{I}_{wm,wm} \end{pmatrix},$$

where

$$\hat{I}_{bm,bm} = \sum_{\substack{i=1\\ \Delta_{ij}=1}}^{n}\sum_{j=1}^{n_i}\hat{I}_{bijm,bijm}$$
$$= \sum_{\substack{i=1\\ \Delta_{ij}=1}}^{n}\sum_{j=1}^{n_i}\frac{(\sum_{l:Y_{i_l,j_l}\geq Y_{ij}} G^2_{bi_lm}\theta_{i_lj_lm})(\sum_{l:Y_{i_l,j_l}\geq Y_{ij}}\theta_{i_lj_lm}) - (\sum_{l:Y_{i_l,j_l}\geq Y_{ij}} G_{bi_lm}\theta_{i_lj_lm})^2}{(\sum_{l:Y_{i_l,j_l}\geq Y_{ij}}\theta_{i_lj_lm})^2},$$

$$\hat{I}_{wm,wm} = \sum_{\substack{i=1\\ \Delta_{ij}=1}}^{n}\sum_{j=1}^{n_i}\hat{I}_{wijm,wijm}$$
$$= \sum_{\substack{i=1\\ \Delta_{ij}=1}}^{n}\sum_{j=1}^{n_i}\frac{(\sum_{l:Y_{i_l,j_l}\geq Y_{ij}} G^2_{wi_lj_lm}\theta_{i_lj_lm})(\sum_{l:Y_{i_l,j_l}\geq Y_{ij}}\theta_{i_lj_lm}) - (\sum_{l:Y_{i_l,j_l}\geq Y_{ij}} G_{wi_lj_lm}\theta_{i_lj_lm})^2}{(\sum_{l:Y_{i_l,j_l}\geq Y_{ij}}\theta_{i_lj_lm})^2},$$

and

$$\hat{I}_{bm,wm} = \hat{I}_{wm,bm} = \sum_{\substack{i=1\\ \Delta_{ij}=1}}^{n}\sum_{j=1}^{n_i}\hat{I}_{bijm,wijm} = \sum_{\substack{i=1\\ \Delta_{ij}=1}}^{n}\sum_{j=1}^{n_i}\hat{I}_{wijm,bijm}$$
$$= \sum_{\substack{i=1\\ \Delta_{ij}=1}}^{n}\sum_{j=1}^{n_i}\Big[\frac{(\sum_{l:Y_{i_l,j_l}\geq Y_{ij}} G_{wi_lj_lm}G_{bi_lm}\theta_{i_lj_lm})(\sum_{l:Y_{i_l,j_l}\geq Y_{ij}}\theta_{i_lj_lm})}{(\sum_{l:Y_{i_l,j_l}\geq Y_{ij}}\theta_{i_lj_lm})^2}$$
$$- \frac{(\sum_{l:Y_{i_l,j_l}\geq Y_{ij}} G_{wi_lj_lm}\theta_{i_lj_lm})(\sum_{l:Y_{i_l,j_l}\geq Y_{ij}} G_{bi_lm}\theta_{i_lj_lm})}{(\sum_{l:Y_{i_l,j_l}\geq Y_{ij}}\theta_{i_lj_lm})^2}\Big].$$

Under the null hypothesis $\gamma_w = 0$, we can define the efficient score of γ_{wm} for the mth variant by removing the effects of the nuisance parameters as:

$$S_{wijm} = U^0_{wijm} - \hat{I}^0_{wijm,bijm}(\hat{I}^0_{bijm,bijm})^{-1}U^0_{bijm},$$

where U^0_{wijm} and U^0_{bim} are the score statistics for within- and between-family components under the null hypothesis. $\hat{I}^0_{wijm,bim}$ and $\hat{I}^0_{bim,bim}$ are components of the observed Fisher information matrix under the null hypothesis. This efficient scores of rare variants were used to construct the burden and kernel tests.

Let S_w be an $n \times n_i \times k$ matrix of efficient scores with $S_w[i,j,m] = S_{wijm}$, construct $\tilde{S}_w$ as an $N \times k$

matrix of efficient scores where $\tilde{S}_w = (S_w[1,\cdot,\cdot]^T, \ldots, S_w[n,\cdot,\cdot]^T)^T$, and W is a $k \times k$ diagonal weight matrix with $W[m,m] = w_m$ being the weight of the mth variant. Also let $\mathbf{1}_N$ be a vector of N 1s and $\mathbf{1}_k$ be a vector of k 1s. The test statistic of the burden test, FamBAC, is formed as:

$$\begin{aligned} T_{FamBAC} &= \frac{(\sum_{i=1}^{n}\sum_{j=1}^{n_i}\sum_{m=1}^{k} w_m S_{wijm})^2}{\sum_{i=1}^{n}\sum_{j=1}^{n_i}(\sum_{m=1}^{k} w_m S_{wijm})^2} \\ &= \frac{(\tilde{S}_w^T \mathbf{1}_N)^T W \mathbf{1}_k \mathbf{1}_k^T W (\tilde{S}_w^T \mathbf{1}_N)}{(\tilde{S}_w^T W \mathbf{1}_k)^T (\tilde{S}_w^T W \mathbf{1}_k)}. \end{aligned}$$

We can prove that all elements of S_w are asymptotically distributed as a normal distribution with mean 0 under the null hypothesis. It is also reasonable to assume that under the null hypothesis, the efficient scores of the offsprings in each family are independently identically distributed. Then, according to the central limit theorem, T_{FamBAC} is asymptotically distributed as χ^2 distribution with degree of freedom 1.

Similarly, the test statistic of the kernel test FamKAC can be written as:

$$\begin{aligned} T_{FamKAC} &= \sum_{m=1}^{k} w_m^2 (\sum_{i=1}^{n}\sum_{j=1}^{n_i} S_{wijm})^2 \\ &= (\tilde{S}_w^T \mathbf{1}_N)^T W^2 (\tilde{S}_w^T \mathbf{1}_N). \end{aligned}$$

It can be proven that under the null hypothesis, all elements of S_w are asymptotically distributed as a normal distribution with mean 0 under the null hypothesis, and $\tilde{S}_w^T \mathbf{1}_N = (\sum_{i=1}^{n}\sum_{j=1}^{n_i} S_{wij1}, \ldots, \sum_{i=1}^{n}\sum_{j=1}^{n_i} S_{wijk})^T$ is asymptotically distributed as a multivariate normal distribution with mean $\mathbf{0}$ and covariance matrix $Ncov(S_{w1}, S_{w2}, \ldots, S_{wk})$, where $S_{w1}, S_{w2}, \ldots, S_{wk}$ are the efficient scores of the k variants, computed from S_w. Therefore, T_{FamKAC} can be approximated by a mixture of χ^2 distributions, that is,

$$T_{FamKAC} \sim \sum_{m=1}^{k} \lambda_m \chi^2(1),$$

where $\lambda_1, \ldots, \lambda_k$ are the eigenvalues of $V = NW^2 cov(S_{w1}, S_{w2}, \ldots, S_{wk})$. Davies exact method [3] can then be used to approximate the mixture of χ^2 distribution and compute p-value.

References

1. Ripatti S, Palmgren J. Estimation of multivariate frailty models using penalized partial likelihood. Biometrics. 2000;56(4):1016–1022.

2. Therneau TM, Grambsch PM, Pankratz VS. Penalized survival models and frailty. Journal of computational and graphical statistics. 2003;12(1):156–175.

3. Davies RB. The distribution of a linear combination of x2 random variables. Applied Statistics. 1980;29(3):323–333.

4. Lee S, Wu MC, Lin X. Optimal tests for rare variant effects in sequencing association studies. Biostatistics. 2012;13(4):762–75. doi:10.1093/biostatistics/kxs014.
