# Supplementary material for: Family-based association tests for rare variants with censored traits
Source: PLoS One. 2019 Jan 25;14(1):e0210870. doi: 10.1371/journal.pone.0210870 (PMC6347269; doi:10.1371/journal.pone.0210870)
Supplement: S2 Appendix — (PDF) [file pone.0210870.s002.pdf]

S2 Appendix: Observed Fisher information matrix for parent-offspring trios

The observed Fisher information matrix, specifically for the simple case of parent-offspring trios, can be written as:

$$\hat{I}_p = \begin{pmatrix} \hat{I}_{bm,bm} & \hat{I}_{bm,wm} \\ \hat{I}_{wm,bm} & \hat{I}_{wm,wm} \end{pmatrix}$$

where

$$\hat{I}_{bm,bm} = \sum_{i:\Delta_i=1} \hat{I}_{bim,bim} = \sum_{i:\Delta_i=1} \frac{(\sum_{j:Y_j \geq Y_i} G_{bjm}^2 \theta_{jm})(\sum_{j:Y_j \geq Y_i} \theta_{jm}) - (\sum_{j:Y_j \geq Y_i} G_{bjm}\theta_{jm})^2}{(\sum_{j:Y_j \geq Y_i} \theta_{jm})^2},$$

$$\hat{I}_{wm,wm} = \sum_{i:\Delta_i=1} \hat{I}_{wim,wim} = \sum_{i:\Delta_i=1} \frac{(\sum_{j:Y_j \geq Y_i} G_{wjm}^2 \theta_{jm})(\sum_{j:Y_j \geq Y_i} \theta_{jm}) - (\sum_{j:Y_j \geq Y_i} G_{wjm}\theta_{jm})^2}{(\sum_{j:Y_j \geq Y_i} \theta_{jm})^2},$$

and

$$\begin{aligned}\hat{I}_{bm,wm} = \hat{I}_{wm,bm} &= \sum_{i:\Delta_i=1} \hat{I}_{bim,wim} = \sum_{i:\Delta_i=1} \hat{I}_{wim,bim} \\ &= \sum_{i:\Delta_i=1} \frac{(\sum_{j:Y_j \geq Y_i} G_{wjm}G_{bjm}\theta_{jm})(\sum_{j:Y_j \geq Y_i} \theta_{jm}) - (\sum_{j:Y_j \geq Y_i} G_{wjm}\theta_{jm})(\sum_{j:Y_j \geq Y_i} G_{bjm}\theta_{jm})}{(\sum_{j:Y_j \geq Y_i} \theta_{jm})^2},\end{aligned}$$

where $\theta_i = \exp(X_i + G_{bi}\gamma_b + G_{wi}\gamma_w + Q_i)$ and $i = 1, \ldots, n$.
