# Supplementary material for: Family-based association tests for rare variants with censored traits
Source: PLoS One. 2019 Jan 25;14(1):e0210870. doi: 10.1371/journal.pone.0210870 (PMC6347269; doi:10.1371/journal.pone.0210870)
Supplement: S3 Appendix — (PDF) [file pone.0210870.s003.pdf]

S3 Appendix: Parameter settings for COSI in simulation studies

Simulation Parameters

length 30000

mutation_rate $1.5e - 8$

recomb_file model.test

gene_conversion_rate $4.5e - 9$

pop_define 1 european

pop_define 3 african-american

pop_define 4 asian

pop_define 5 african

pop_size 1 100000

sample_size 1 20000

pop_size 3 100000

sample_size 3 0

pop_size 4 100000

sample_size 4 0

pop_size 5 100000

sample_size 5 0

pop_event migration_rate "afr->eur migration" 5 1 0. .000032

pop_event migration_rate "eur->afr migration" 1 5 0 .000032

pop_event migration_rate ”afr->as migration” 5 4 0. .000008

pop_event migration_rate ”as->afr migration” 4 5 0 .000008

pop_event admix ”african american admix” 3 1 5. .2

pop_event split ”african to aa” 5 3 7.0

pop_event change_size ”agriculture - african” 5 200 24000

pop_event change_size ”agriculture - european” 1 350 7700

pop_event change_size ”agriculture - asian” 4 400 7700

pop_event bottleneck ”african bottleneck” 5 1997 .008

pop_event bottleneck ”asian bottleneck” 4 1998 .067

pop_event bottleneck ”european bottleneck” 1 1999 .02

pop_event split ”asian and european split” 1 4 2000

pop_event migration_rate ”afr->eur migration” 5 1 1996 0

pop_event migration_rate ”eur->afr migration” 1 5 1995 0

pop_event migration_rate ”afr->as migration” 5 4 1994 0

pop_event migration_rate ”as->afr migration” 4 5 1993 0

pop_event bottleneck ”OoA bottleneck” 1 3499 .085

pop_event split ”out of Africa” 5 1 3500

pop_event change_size ”african pop size” 5 17000 12500

Recombination file: model.test

1 $1.02527e-09$

7761 $3.08484e-09$

7762 $1.02527e-09$

12547 $7.47647e-09$

12548 $1.02527e-09$

20987 $1.08700e-09$

20988 $1.02527e-09$

25058 $2.57265e-09$

$25059\ 1.02527e-09$

$27393\ 1.04397e-09$

$27394\ 1.02527e-09$
